# Australian clinicians and chemoprevention for women at high familial risk for breast cancer

**DOI:** 10.1186/1897-4287-7-9

**Published:** 2009-05-04

**Authors:** Louise A Keogh, John L Hopper, Doreen Rosenthal, Kelly-Anne Phillips

**Affiliations:** 1 Key Centre for Women's Health in Society, School of Population Health, University of Melbourne, Australia; 2 Centre for MEGA Epidemiology, School of Population Health, University of Melbourne, Australia; 3 Division of Haematology and Medical Oncology, Peter MacCallum Cancer Centre, Australia; 4 Department of Medicine, St Vincent's Hospital, The University of Melbourne, Australia

## Abstract

**Objectives:**

Effective chemoprevention strategies exist for women at high risk for breast cancer, yet uptake is low. Physician recommendation is an important determinant of uptake, but little is known about clinicians' attitudes to chemoprevention.

**Methods:**

Focus groups were conducted with clinicians at five Family Cancer Centers in three Australian states. Discussions were recorded, transcribed and analyzed thematically.

**Results:**

Twenty three clinicians, including genetic counselors, clinical geneticists, medical oncologists, breast surgeons and gynaecologic oncologists, participated in six focus groups in 2007. The identified barriers to the discussion of the use of tamoxifen and raloxifene for chemoprevention pertained to issues of evidence (evidence for efficacy not strong enough, side-effects outweigh benefits, oophorectomy superior for mutation carriers), practice (drugs not approved for chemoprevention by regulatory authorities and not government subsidized, chemoprevention not endorsed in national guidelines and not many women ask about it), and perception (clinicians not knowledgeable about chemoprevention and women thought to be opposed to hormonal treatments).

**Conclusion:**

The study demonstrated limited enthusiasm for discussing breast cancer chemoprevention as a management option for women at high familial risk. Several options for increasing the likelihood of clinicians discussing chemoprevention were identified; maintaining up to date national guidelines on management of these women and education of clinicians about the drugs themselves, the legality of "off-label" prescribing, and the actual costs of chemopreventive medications.

## Introduction

Effective chemoprevention strategies are now available for women at increased risk for breast cancer [1]. This represents a major addition to the risk management options for such women, particularly those who choose to avoid or postpone risk-reducing surgery. However, uptake of breast cancer chemoprevention is low in women at high familial risk [2-5]. The reasons for this low uptake must be elucidated with priority if the promise of chemoprevention to reduce morbidity from breast cancer is to be fulfilled.

Four randomized controlled trials involving over 25,000 women at increased risk have demonstrated that tamoxifen taken daily for 5 years reduces breast cancer risk by 30–50% [6-9]. The preventive effect is sustained for at least five years after cessation of therapy [9] and the risk of serious side-effects is low, particularly for premenopausal women [10,11]. Raloxifene is an alternative option for postmenopausal women. It is as effective as tamoxifen in preventing invasive breast cancer somewhat less effective in preventing in situ cancers [12] and has a superior side-effect profile particularly with regard to gynaecological side-effects and thrombosis. Both drugs predominantly reduce incidence of estrogen receptor positive tumors.

There are no data on the potential efficacy of Raloxifene for chemoprevention in BRCA1 and BRCA2 mutation carriers and limited data for tamoxifen. Breast cancers arising in BRCA1 mutation carriers are estrogen receptor negative in 80–90% of cases, conversely those arising in BRCA2 mutation carriers are usually estrogen receptor positive [3] In the NSABP-P1 chemoprevention trial [13] the estimated risk ratios for BRCA1 and BRCA2 mutation carriers who took tamoxifen versus placebo were 1.67 (95% confidence interval 0.32–10.7) and 0.38 (95% confidence interval 0.06–1.56) respectively. The number of mutation carriers who developed breast cancer in this study were very small (8 BRCA1 and 11 BRCA2), so the confidence intervals are wide and the results difficult to interpret. However a retrospective study demonstrated a 50% reduction in risk of contralateral breast cancer for both BRCA1 (odds ratio = 0.50: 95% CI, 0.30–0.85) and BRCA2 (odds ratio = 0.42: 95% CI, 0.17–1.02) mutation carriers who took tamoxifen after their first breast cancer. Also in that study, the protective effect of tamoxifen was not seen for mutation carriers who had undergone oophorectomy (odds ratio = 0.83: 95%CI, 0.24–2.89), but that subgroup was small and so the question of whether tamoxifen confers additional protection against breast cancer in mutation carriers who have undergone oophorectomy remains controversial.

The type of provider whom patients see for genetic testing has been found to contribute to variations in prophylactic treatment [14]. Physician recommendation is an important determinant of uptake of breast cancer chemoprevention[15,16]. Whether a physician informs the client of the option of chemoprevention, the strength of the recommendation [17] and the framing of the information [18] might all be expected to influence uptake of chemoprevention. Although surveys of physicians to determine chemoprevention prescribing rates have been conducted in North America, few examined the reasons for the low rates [19,20]. Qualitative method is particularly suited to answering this question as the variables influencing prescribing have not yet been identified [21].

In Australia, there is a network of Family Cancer Centers (FCCs), funded by State governments, that does the vast majority of assessment and genetic testing of women at high familial risk for breast cancer. A prior Australian study undertaken between 1998 and 2000, demonstrated that FCC clinicians discussed chemoprevention in 58% of pre-genetic testing consultations, although most discussions (41%) were focused on a chemoprevention trial [22]. The purpose of the current study was to identify and explore the contemporary barriers and enablers to clinicians discussing and/or recommending chemoprevention in their client consultations in the setting of FCCs. Focus group method was chosen as it was more likely to reveal areas of shared understanding and areas of dispute among FCC clinicians than individual interviews and is more reflective of the multidisciplinary approach to care used in the clinics. Focus groups also create a more relaxed environment for participants as the group is under study rather than the individual [21].

## Methods

The study population was clinicians working in any one of five FCCs in one of three Australian capital cities. In the two cities in which there was more than one Center operating, the two largest Centers were selected. Clinicians, including clinical geneticists, genetic counselors, medical oncologists, breast surgeons, breast physicians and gynaecologic oncologists, were eligible for the study if they provided risk management advice to women at high risk for breast cancer. A contact person at each Center, usually the Director, determined the eligibility of the clinicians at that Center. For some Centers, genetic counselors were considered eligible, while for others, only physicians were eligible. The decision depended on clinic protocols for the provision of risk management advice, and was not questioned by the researchers.

**Figure 1:**
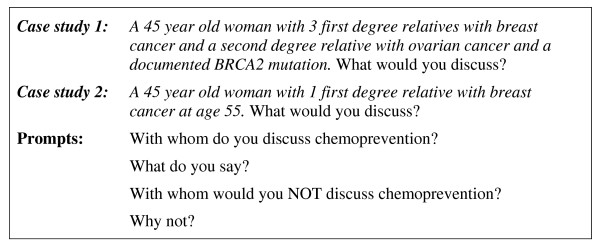
**Hypothetical scenarios and an example of prompts used in focus group discussions**.

The time and location of the focus groups was negotiated with the contact person at each Center. A participant information sheet, and invitation to a focus group on the provision of risk management advice was e-mailed to eligible clinicians by the contact person. Focus groups were facilitated by LK, using a list of prompts and two case studies (Figure 1). Initially, participants were asked to describe the FCC, the role of clinicians, and the average client and their pathway through the FCC and beyond. Case studies were then used to identify more specific information about risk management and each intervention was discussed. The discussion was tape recorded, transcribed, de-identified and analyzed thematically.

Transcripts were read to identify the main themes. All relevant data was then coded under one or more of the themes and each theme analyzed by two researchers (KAP & LK) to ensure reliability. The study was approved by the Human Research and Ethics Committees (HREC) at each recruiting institution. Written informed consent from the participating clinicians was only considered necessary by the HREC at one site. For the other sites, attendance at the focus group session was considered to represent implied consent.

## Results

Twenty three of 36 eligible clinicians participated in six focus groups, (3 to 5 participants per group) between April and August 2007. Participant characteristics are summarized in Table 1. Two thirds of participants were women, and a range of specialties were represented.

**Table 1:** Characteristics of participants

**Specialty**	**Number**
Genetic Counselor	4

Clinical Geneticist	5

Medical Oncologist/Fellow	10

Breast Physician/Surgeon	3

Gynecologic Oncologist	1

	

**Gender**	

Male	7

Female	16

Three main codes were identified from the data; 1) 'Perception of role' (how clinicians described their role in the management of high risk women) 2) 'Case study one' (all discussion in response to case study one), 3) 'Chemoprevention' (all discussion of chemoprevention either in response to case studies or to any other question, including the specific question "with whom do you discuss chemoprevention?"). The 'Case study one' and 'Chemoprevention' codes were analyzed for this report. The analysis identified both the similarities and differences in response to case study one, and the barriers and enablers to the discussion of chemoprevention.

### Case Study 1

Participants discussed how they would advise the woman in Case Study 1. Prior to tailoring a risk management strategy, participants said they would outline the options available. The range of options potentially discussed were screening (mammography, MRI, ultrasound, clinical breast exam), lifestyle modifications, surgery (mastectomy and bilateral salpingo-oophorectomy (BSO)) and/or chemoprevention (Tamoxifen or Raloxifene). Mostly, clients leave the FCC with a personalised strategy which is discussed with them, and reiterated in a subsequent letter. Clients are then expected to execute their risk management strategy either through regular attendance at a Risk Management Clinic (only available in selected Centers) [23], or through their family doctor or specialist.

**Figure 2:**
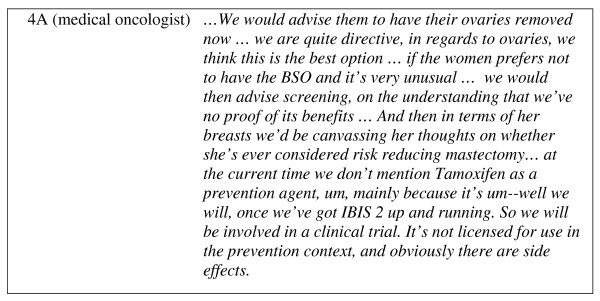
**Example of a response to Case Study 1**.

The process of devising a management strategy was summarised by 2C (breast surgeon): '*All of these things are on the table at the first instance, for discussion. Then people will make up their mind down which path they want to travel'*. In most Centers however, the clinicians would recommend a preferred strategy. In each Center, the preferred strategy for Case Study 1 was BSO (Figure 2). Clinicians from 3 of 5 Centers mentioned chemoprevention in the discussion of options for management of Case Study 1, but clinicians from 2 Centres stated explicitly that they would not mention it as an option (Figure 2).

### Potential candidates for chemoprevention

Focus groups who said they did mention chemoprevention were asked who they discussed it with. Groups 1, 2, 5 and 6 identified mutation carriers, but particularly BRCA2 carriers. 1D (medical oncologist) was the only participant to suggest that women at high risk without a documented mutation should also be informed about chemoprevention, with the proviso of '*explaining where the uncertainty still lies*'. Conversely 6D (medical oncologist) said '*BRCA mutation carriers, particularly BRCA2, I'm not too sure about high risk women without BRCA mutation ... I would be more comfortable with just the mutation carrier. I mean, if the high risk woman raised that I would discuss that...but it may not be our recommendation to jump in ... and just take it.'*.

### Barriers to Discussing Breast Cancer Chemoprevention

Analysis of the code 'Chemoprevention' revealed several barriers and enablers to the discussion of chemoprevention with women at high risk of breast cancer (Tables 2 and 3). Each of the barriers related either to the available evidence for the effectiveness of chemoprevention, practical constraints to discussing it with women, or to perceptions held by clinicians about chemoprevention.

**Table 2:** Barriers to the discussion of breast cancer chemoprevention in Family Cancer Centres with supporting quotes

EVIDENCE	Participant	No	Quote
Evidence not strong enough	1A (med oncologist)	1	*I don't think that there's really strong evidence to support a reduction in breast cancer occurrence *[in BRCA1 and BRCA2 mutation carriers].
	4A (med oncologist)	2	*It may be effective in BRCA2 carriers, but the numbers are very small and so, we probably need some further evidence*.
	5C (med oncologist)		*There are no randomized trials in high risk women, truly high risk women*
	1C (med oncologist)	3	*There's no survival benefit*.
	2B (gynae oncologist)	4	*You may... be preventing some cancers but you may not be saving lives*.

Side effects outweigh benefits	2A (breast surgeon)	5	*The data would support that there is risk reduction but... at a price, of the side effects and complications and I don't personally think that that sort of balance is such to encourage people to be taking the medication*.

BSO superior to chemoprevention in BRCA mutation carriers	5B (genetic counsellor)	6	*And next the question that I don't know, and I don't think anyone does, is what's the benefit of Tamoxifen in addition to the benefit you're going to get from oophorectomy*...
	4A (med oncologist)	7	*Because of the very high uptake – 70%, of BSOs, it doesn't routinely come up*.

PRACTICE			

Not TGA approved* for prevention	2C (gynae oncologist)	8	..*and also because it [*Tamoxifen*] is not approved in Australia for prevention, you have to pay for it..... It's not hugely expensive but it's not cheap. And you can tell people that it is approved in the USA for this purpose but not in Australia, and you can give them the facts*.

Not on the PBS** for prevention	3A (clinical geneticist)	9	*When the IBIS report showed Tamoxifen reduced the risk of breast cancer, we kept a watching brief on that as it were, but didn't pursue it in detail because Tamoxifen was not and is still not on the PBS*.

Chemoprevention only referred to in national guidelines as part of clinical trials	4A (med oncologist)	10	*We discuss it when the chemoprevention trials are open..... But.... once the IBIS I closed, we have not routinely been discussing chemoprevention with the unaffected woman*.
	5A (clinical geneticist)	11	*If you go back to all of the management guidelines and look at the...recommendations for chemoprevention, there's very few of them that actually recommend it*.

Not many women ask about it	1D (med oncologist)	12	*Up until recently I haven't really delved into it ... unless the woman has appeared interested in some way*.
	1C (med oncologist)	13	*Not many woman come forward asking questions about this*.

**PERCEPTION**	**Participant**	**No**	**Quote**

(We are) not knowledgeable about chemoprevention	3B (clinical geneticist)	14	*Chemoprevention is talked about very little because basically we have very little knowledge of that' *and later, '*if a woman specifically asks that question *[I]*would say, look that's not my area, go back to your oncologist*.
	Dialogue between 6E (clinical geneticist) and 6D (med oncologist)	15	*LK* *Do you discuss it with people, 6E?* *6E* *Look, pretty infrequently, that's the sort of thing that um, well if it does crop up it's usually because I've dragged 6D in to see my patient *[laughs]. *LK* *And is that because of your specialty 6D?* *6D* *I'm an oncologist. So I would be more comfortable to discuss what the risks are, what the benefits are of, you know, taking Tamoxifen*.

Women are opposed to hormonal treatment	1D (med oncologist)	16	*This group as a whole are not really using any form of hormonal intervention, whether it be the pill, HRT or Tamoxifen, they are un-keen*. And later: [They have had it] *drummed into them right from the year dot, hormones are bad, hormones are bad*.

* The Australian Therapeutic Goods Administration (TGA) has a similar role to the U.S. Federal Drugs Administration

**The Australian Pharmaceutical Benefits Scheme (PBS) provides subsidized pharmaceuticals to all Australians

**Table 3:** Enablers to the discussion of chemoprevention in Family Cancer Centres with supporting quotes

EVIDENCE	Participant	No	Quote
'Reasonable' evidence in BRCA2 carriers	5C (med oncologist)	17	*I think that in a BRCA2 mutation carrier we'll discuss Tamoxifen- I'm a bit more confident about Tamoxifen with a BRCA2, than in a BRCA1*.
Side-effects less in younger women	1A (med oncologist)	18	...*side effects at that age *[45] *are likely be to be small in absolute terms*
PRACTICE			

Enroling people on a trial is convenient	1B (med oncologist)	19	*When the IBIS I study was recruiting that would be definitely part of my discussion because there was a study that they could participate in to try and get an answer. But then that closed so there's been a window between now and then [-]... There's sort of been a bit of a lull*.

Not expensive	5C (med oncologist)	20	identified that not being PBS-listed was not a constraint to prescribing Tamoxifen because it '*is not an expensive drug'*

PERCEPTION			

Action of Tamoxifen to reduce risk 'intuitively makes sense'	5C (med oncologist)	21	*But intuitively, I can understand how um, Tamoxifen would reduce the risk*.
Lack of 'hard data' is not always a barrier to recommending something 5C (med oncologist)	5A (clinical geneticist)	22	*5A Breast ultrasound? If you're having MRI? No, but if you aren't having it, then, there's very little hard data*... *5C I know there's no hard data but that's never stopped anyone doing anything*

#### Evidence

Some felt the evidence supporting the effectiveness of chemoprevention was not strong enough to justify recommending it to their clients and the evidence was seen as weak in several different ways (quotes 1–4, Table 2).

Another issue related to evidence was the perceived adverse balance between benefits and side effects (quote 5, Table 2).

Quotes 6 and 7, table 2 demonstrate that another common barrier regarding evidence was the perception that BSO was more effective than chemoprevention at reducing breast cancer risk in mutation carriers.

#### Practice

Clinicians also identified a number of barriers to the discussion of chemoprevention that were more practical. The fact neither Tamoxifen nor Raloxifene is licensed in Australia for use in chemoprevention and neither is listed for chemoprevention on the Pharmaceutical Benefits Scheme (PBS) acted as barriers among this group of clinicians (quotes 8 and 9, table 2). Another barrier was that chemoprevention is included in Australian guidelines on management of high risk women only in terms of offering participation in "a relevant approved clinical trial"[24] (quotes 10 and 11, table 2).

The fact that women rarely asked about chemoprevention also acted as a barrier for clinicians. Some clinicians relied on women's interest to guide their discussion, as evidenced by the comments made by two medical oncologists in Group 1 (quotes 12 and 13, table 2)

#### Perception

Clinicians' perception that they were personally not knowledgeable about chemoprevention was a further barrier to recommending it to their clients (quote 14, table 2). This particular barrier was strongly influenced by the clinician specialty, with oncologists expressing more confidence in their knowledge about Tamoxifen than clinical geneticists, as demonstrated during the exchange in quote 15, Table 2 between a clinical geneticist and medical oncologist.

Clinicians also reported that women's perceptions acted as barriers to discussing chemoprevention, specifically negative attitudes towards hormonal treatments in general, and chemoprevention in particular (quote 16, table 2).

### Enablers to Discussing Chemoprevention

Analyzing the few instances where chemoprevention was discussed favorably or even recommended to clients by clinicians, it was possible to identify the things that enabled the clinician to take this stance. Table 3 summarizes the three categories of enablers, related to either evidence, practice or perception.

#### Evidence

Those who recommended Tamoxifen felt that there was reasonable evidence to support its use as a chemopreventive agent for BRCA2 mutation carriers (quote 17, table 3). One clinician (medical oncologist) also identified that the risk/benefit ratio is more favorable in younger women (quote 18, table 3).

#### Practice

A practical enabler to providing chemoprevention was being able to offer clients enrolment in a trial. As shown earlier, one of the medical oncologists was comfortable placing clients on a research trial (quote 10, table 2). This position is also consistent with the current national guidelines. A similar sentiment was expressed by another medical oncologist (quote 19, table 3).

The fact that tamoxifen is not an expensive drug was also identified as an enabler to prescribing (quote 20, table 3).

#### Perception

5C perceived the action of Tamoxifen to reduce the risk of breast cancer for BRCA2 carriers as something that clinicians '*intuitively understand'*, and acknowledged that the lack of '*hard data' *had '*never stopped anyone doing anything' *(quotes 21 and 22, Table 3). This perception is counter to the perception expressed by others, namely that the evidence is not good enough, and that lack of evidence is a barrier.

## Discussion

Despite high quality evidence of the efficacy of Tamoxifen and Raloxifene for breast cancer prevention, international uptake, even among very high risk women, is much lower than anticipated. The potential of breast cancer chemoprevention to improve morbidity is hence not fully realized. The situation is also jeopardizing future advances in the area. The planned NSABP-P4 trial which was to compare the efficacy of Raloxifene and Letrozole in this setting has been postponed indefinitely because, according to the Director of the U.S. National Cancer Institute, previous studies "while scientifically enlightening, have failed to change the practice of breast cancer prevention among women and their healthcare providers"[25]. It is therefore critically important to understand the barriers to the discussion of breast cancer chemoprevention with high risk women. In this study of 23 clinicians in FCCs, comprising 64% of eligible participants and 56% of clinicians who are members of the Australian national consortium for familial breast cancer [26], we identified several barriers and enablers to the discussion of chemoprevention with clients.

Many clinicians felt that the evidence for use of chemoprevention was not strong enough for them to discuss it with their clients. While there is clear evidence from large randomized controlled trials that Tamoxifen reduces breast cancer risk by 30–50% [6-9], clinicians expressed concern about how applicable these findings were to the population of women that they see. Clinicians in this study were also concerned that because a survival benefit has not been demonstrated, the evidence was inadequate, yet they routinely discuss mammographic and MRI screening and risk-reducing mastectomy with their clients, strategies where no survival benefit has been shown for this group.

Like the clinicians studied by Peshkin, clinicians in our study were more likely to discuss chemoprevention with a BRCA2, rather than a BRCA1, mutation carrier [19]. BRCA1 carriers (unlike BRCA2 carriers) usually develop estrogen receptor alpha negative tumors and the randomized chemoprevention studies have all failed to demonstrate a decrease in such tumors. However, estrogen may be important in the pathogenesis of BRCA1-associated BC [27] and tamoxifen and raloxifene may act as preventive agents in BRCA1 carriers by binding to estrogen receptor beta [28]. BSO, a strategy embraced by many of the clinicians in the study, reduces incidence of BRCA1-associated breast cancer also presumably by a hormonal mechanism.

Of particular interest, clinicians in this study were more comfortable discussing chemoprevention with BRCA2 carriers than high risk women without a demonstrated mutation. The reason for this was unclear from the focus group discussions, especially since BSO would not usually be recommended to reduce breast cancer risk in the latter group and there is stronger evidence for efficacy of chemoprevention in that group. Perhaps it is because high-risk women without an identified mutation are, on average, at lower lifetime risk for breast cancer (30–40%) than BRCA1 and BRCA2 mutation carriers (60–80%) and thus the balance of risks and benefits is assessed differently.

Potential side-effects of chemoprevention are an important issue. The perception that side-effects outweighed the benefits was expressed by clinicians in this study. Vasomotor and gynaecologic symptoms of women on chemoprevention are mostly manageable [8,9] or resolve on cessation of therapy, but endometrial cancer and thrombosis are potentially serious side-effects. Endometrial cancer risk is not increased for pre-menopausal women, and for post-menopausal women the risk is less with Raloxifene compared with Tamoxifen. The excess risk of endometrial cancer and thrombotic events respectively have been estimated at 1 in 2686 and 1 in 1042 per year of preventive Tamoxifen use [11]. Some, but not all, women will consider these risks tolerable in the setting of a 30–50% reduction in their breast cancer risk. We argue that clinicians should not make assumptions about what their clients will accept. Instead women should be routinely informed of the risks and benefits of chemoprevention along with the other available risk management strategies.

Similarly, clinicians cited their clients' lack of knowledge about chemoprevention as a barrier to discussing it with them, but until chemoprevention is discussed as an option, it is unlikely that awareness of chemoprevention will be increased among those eligible to use it.

The fact that Tamoxifen and Raloxifene are not licensed by the Australian Therapeutic Goods Administration (TGA) and not listed on the Australian PBS for the specific indication of breast cancer prevention was perceived as a barrier to discussing and prescribing chemoprevention. The Australian TGA grants marketing approval for specific medicines for specific indications, but once a medicine is approved for one indication (e.g. treatment of breast cancer) it *can *be prescribed 'off-label' for another. Off-label prescribing is an accepted and common practice in oncology [29,30]. Our study suggests that perhaps not all clinicians who advise high risk women are aware of off-label prescribing, specifically, those who do little or no prescribing, such as clinical geneticists and genetic counselors. Alternatively, the TGA and PBS non-listed status of Tamoxifen and Raloxifene for chemoprevention may contribute to the idea that their use in this setting is not legitimate, rather than acting as barriers directly: in Australia, Raloxifene costs approximately $2.25 per day, and Tamoxifen $0.20 to $1 per day.

Off-study use of chemoprevention in this setting is supported in international guidelines [31-33], but not Australian guidelines[24]. Our study identified this as a barrier to its use, highlighting the importance of keeping guidelines up to date. The Australian guidelines were published in 1999 and do not incorporate any of the evidence from trials published after that date, including IBIS I [9,34]. Updated Australian guidelines, which include use of chemoprevention as a management option have been prepared by the authors and others but are not yet published. Their publication and dissemination may address several of the barriers identified in the study.

In a study of Californian physicians, 40% indicated that they did not have time to adequately discuss breast cancer risk reduction options [20], but in our study lack of time was not identified as a potential barrier.

This study examined the attitudes of clinicians who work in multidisciplinary FCCs around Australia and is likely to be representative of the attitudes of clinicians working in that setting. Although a woman's initial risk assessment would generally occur in such a Center, many women receive their subsequent management from external breast surgeons. It would be of interest to determine the relative importance of the various barriers identified in this and other studies among those clinicians.

Of the barriers to the discussion of breast cancer chemoprevention identified, those with the most straightforward potential interventions include the maintenance of up-to-date national guidelines on management of high-risk women and education of clinicians about off-label prescribing and the relatively low costs of chemopreventive medications. More education about chemoprevention options for those specialties that advise these women but that are unfamiliar with prescribing these drugs in other settings may also improve the likelihood that chemoprevention will be discussed in the consultation. Nevertheless women will ultimately need to weigh the potential benefit against side-effects, and further studies on the use of decision aids in this setting may be useful [35]. Addressing these factors may result in better acceptance of chemoprevention by clinicians which may translate into improved levels of uptake by high risk women.

## Competing interests

The authors declare that they have no competing interests.

## Authors' contributions

LK participated in the design of the study, conducted focus groups, analysed data, and drafted the manuscript. JH participated in the design of the study and drafting of the manuscript. DR participated in the design of the study, and edited manuscript. KP participated in the design of the study, analysis of the data and drafting of manuscript. All authors read and approved the final manuscript.
